# MRE11 inhibition highlights a replication stress-dependent vulnerability of MYCN-driven tumors

**DOI:** 10.1038/s41419-018-0924-z

**Published:** 2018-08-30

**Authors:** Marialaura Petroni, Francesca Sardina, Paola Infante, Armando Bartolazzi, Erica Locatelli, Francesca Fabretti, Stefano Di Giulio, Carlo Capalbo, Beatrice Cardinali, Anna Coppa, Alessandra Tessitore, Valeria Colicchia, Maria Sahùn Roncero, Francesca Belardinilli, Lucia Di Marcotullio, Silvia Soddu, Mauro Comes Franchini, Elena Petricci, Alberto Gulino, Giuseppe Giannini

**Affiliations:** 1 Center for Life Nano Science@Sapienza, Istituto Italiano di Tecnologia, 00161 Rome, Italy; 2 Department of Molecular Medicine, University La Sapienza, 00161 Rome, Italy; 3 Pathology Research Laboratory, St. Andrea University Hospital, 00189 Rome, Italy; 4 Department of Industrial Chemistry “Toso Montanari”, University of Bologna, 40136 Bologna, Italy; 5 Institute of Cell Biology and Neurobiology, National Research Council, 00015 Monterotondo, Italy; 6 Department of Experimental Medicine, University La Sapienza, 00161 Rome, Italy; 7 Department of Biotechnological and Applied Clinical Sciences, University of L’Aquila, L’Aquila, 67100 Italy; 8 Istituto Pasteur-Fondazione Cenci Bolognetti, 00161 Rome, Italy; 9 Unit of Cellular Networks and Molecular Therapeutic Targets, Regina Elena National Cancer Institite _IRCCS, 00144 Rome, Italy; 10 Department of Biotechnology, Chemistry and Pharmacy, University of Siena, 53100 Siena, Italy; 11 Present Address: Institute of Biology and Molecular Pathology-CNR, 00161 Rome, Italy

## Abstract

MRE11 is a component of the MRE11/RAD50/NBS1 (MRN) complex, whose activity is essential to control faithful DNA replication and to prevent accumulation of deleterious DNA double-strand breaks. In humans, hypomorphic mutations in these genes lead to DNA damage response (DDR)-defective and cancer-prone syndromes. Moreover, MRN complex dysfunction dramatically affects the nervous system, where MRE11 is required to restrain MYCN-dependent replication stress, during the rapid expansion of progenitor cells. MYCN activation, often due to genetic amplification, represents the driving oncogenic event for a number of human tumors, conferring bad prognosis and predicting very poor responses even to the most aggressive therapeutic protocols. This is prototypically exemplified by neuroblastoma, where MYCN amplification occurs in about 25% of the cases. Intriguingly, MRE11 is highly expressed and predicts bad prognosis in MYCN-amplified neuroblastoma. Due to the lack of direct means to target MYCN, we explored the possibility to trigger intolerable levels of replication stress-dependent DNA damage, by inhibiting MRE11 in MYCN-amplified preclinical models. Indeed, either MRE11 knockdown or its pharmacological inhibitor *mirin* induce accumulation of replication stress and DNA damage biomarkers in MYCN-amplified cells. The consequent DDR recruits p53 and promotes a p53-dependent cell death, as indicated by p53 loss- and gain-of-function experiments. Encapsulation of *mirin* in nanoparticles allowed its use on MYCN-amplified neuroblastoma xenografts in vivo, which resulted in a sharp impairment of tumor growth, associated with DDR activation, p53 accumulation, and cell death. Therefore, we propose that MRE11 inhibition might be an effective strategy to treat MYCN-amplified and p53 wild-type neuroblastoma, and suggest that targeting replication stress with appropriate tools should be further exploited to tackle MYCN-driven tumors.

## Introduction

MRE11 is a component of the MRE11/RAD50/NBS1 (MRN) complex, which has essential roles in detecting and repairing DNA double-strand breaks (DSBs) and activation of the DNA damage response (DDR) via ATM^1,2^. Within the complex, the NBS1 and RAD50 moieties mediate nuclear localization and interactions with DNA and protein partners. MRE11 is essential to stabilize the complex allowing its accumulation, and to provide the nuclease activities required for the resection of the broken DNA ends^3,4^.

Hypomorphic MRE11 mutations are responsible for the inherited Ataxia-Telangiectasia-like disorder (ATLD), which shares cellular and clinical phenotypes (including immunodeficiency, sterility, and radiosensitivity) with Ataxia Telangiectasia (A-T) and Nijmegen breakage syndrome (NBS), caused by mutations in the ATM and NBS1 genes, respectively^5,6^. Complete loss of *Mre11, Nbs1*, or *Rad50* leads to early embryonic lethality due to severe proliferation defects in vertebrate cells^7–10^. Appropriate animal models recapitulate the main features of human syndromes and support MRN tumor suppressive function^11–13^, consistent with the increased cancer susceptibility observed in MRN-defective human syndromes.

Similar to other DNA repair proteins, MRE11 also plays a pivotal role in controlling the integrity of DNA replication, preventing the deleterious effects of replication stress (RS)^14–17^. Indeed, an inefficient response to RS seems to contribute to the genesis of developmental disorders of the nervous system, in patients and animal models carrying mutations in MRN genes^18,19^.

MYCN is a member of the MYC family of transcription factors, largely expressed in, and required for, nervous system development^20^. As an oncogene, it is deregulated in several neuronal and non-neuronal tumors of childhood, including neuroblastoma, medulloblastoma, retinoblastoma, astrocytoma, rhabdomyosarcoma, Wilm’s tumor, and in adulthood tumors, such as non-small cell lung cancer and breast cancer (http://www.cancerindex.org/geneweb/MYCN.htm). At least in neuroblastoma, where patients are typically stratified into risk groups based on multiple parameters, *MYCN* amplification (MNA) represents the most relevant and independent negative prognostic factor allowing straightforward patient classification into the high-risk group^21–23^. Despite intense multimodal treatment, MNA neuroblastoma patients often relapse and succumb to their disease^22^, which underscores the need for more effective therapeutic approaches for these children.

MYC proteins promote RS, DNA damage, and DDR by several mechanisms^24–31^. Increased levels of RS have been clearly detected in primary MNA tumors as compared to MYCN single copy (MNSC) samples^31^. Moreover, “DNA repair” is among the most significantly deregulated gene ontology groups in neuroblastomas sharing a MYCN signature^32^. Overall, these data suggest that coping with RS and DNA damage is cogent in these tumors and they are consistent with the knowledge that DDR proteins can be recruited by oncogenes to dampen oncogene-dependent RS, eventually favoring cancer cell survival^33–36^.

We recently showed the MRE11, RAD50, and NBS1 are transcriptionally regulated by MYCN in order to prevent the accumulation of RS-dependent DNA damage during MYCN-driven expansion of cerebellar granule progenitor cells^26^. Whether the MRN complex is essential to prevent the deleterious effects of MYCN-dependent RS also in cancer cells was poorly investigated, so far.

Here, we explored the involvement of MRE11 in neuroblastoma as a model for MYCN-driven tumors and addressed the possibility to target the MRN complex to trigger intolerable levels of RS-dependent DNA damage in MNA/high-risk tumors.

## Results

### MRE11 is overexpressed in MNA neuroblastoma and is essential for MYCN-dependent proliferation

**Fig. 1:**
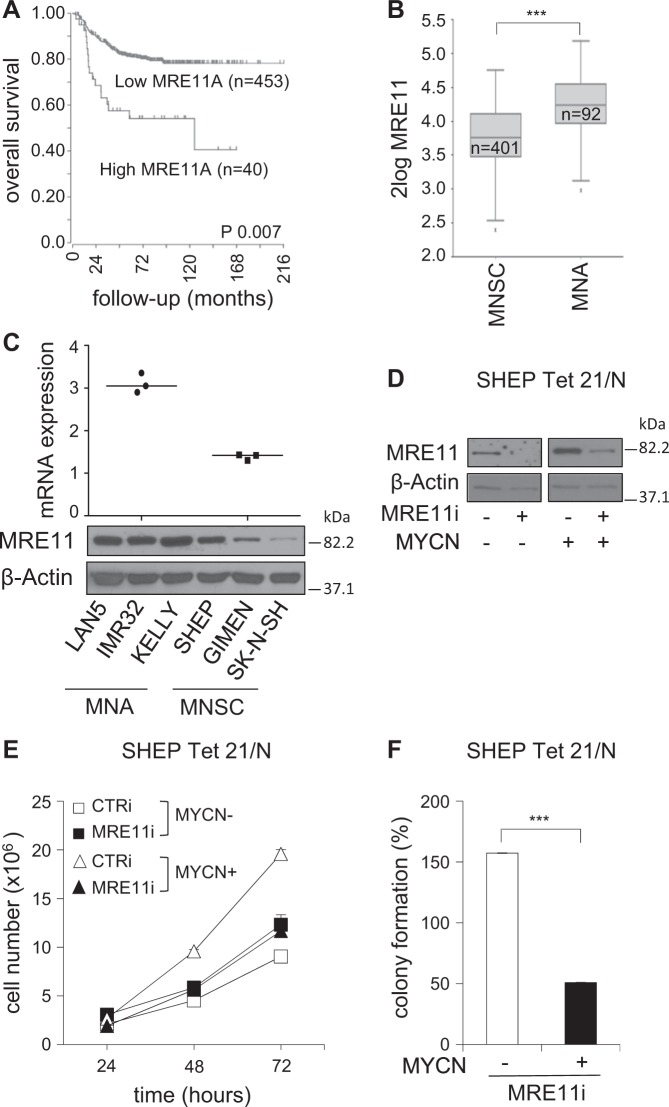
MRE11 expression correlates with poor prognosis and MYCN status in neuroblastoma patients and its depletion affects MYCN-dependent proliferation/survival in vitro. **a** Kaplan–Meier curves reporting patients’ overall survival probability with respect to MRE11 expression. Scan-modus was used for cut-off determination with a minimum group size of 8 to determine the best *p*-values in a log-rank test (high and low expression as defined in Figure S1). *p*-Value was adjusted using Bonferroni correction test for multiple testing. **b** Box plot of MRE11 expression relative to MYCN status. The best *p*-value was calculated using R2-Genomics analysis and visualization platform by the Student’s *t*-test. ***p < 0.001. **c** MRE11 mRNA and protein expression as measured by real-time Q-PCR (upper panel) and western blot (WB) analysis (bottom panel) in MNA (LAN5, IMR32, Kelly) and MNSC (SHEP, GIMEN, SK-N-SH) cell lines. mRNA expression was normalized on GAPDH levels. WBs were probed with β-actin as a loading control. **d**–**f** MYCN+ and MYCN− SHEP Tet21/N cells were subjected to MRE11 knockdown by shRNA interference (MRE11i) and to a short round of puromycin selection. No-target shRNA interference (CTRi) was used as control. Cells were analyzed for MRE11 and β-actin protein expression (**d**) and used for cell proliferation assays (**e**) (data represent mean ± SD of three independent experiments) and colony formation assays (**f**) (average data obtained by three independent experiments are expressed as percentage compared to CTRi-treated controls ± SD). *p* was calculated by the Student’s *t*-test. ***p < 0.001

By interrogating multiple neuroblastoma gene expression datasets on the R2-Genomics platform (http://r2.amc.nl), we noticed that very high MRE11 expression was associated with reduced overall survival in primary human neuroblastoma (Fig. 1a and S1). Consistently, MRE11 mRNA expression was significantly higher in worst prognosis cases characterized by MYCN-amplified (MNA) compared to MNSC neuroblastoma tumors 1 (Fig. 1b). This was further confirmed in neuroblastoma cell lines, at the RNA and protein levels (Fig. 1c). Notably, a very low MRE11 expression was instead associated with poor survival and high-risk and stage, in the MNSC subgroup (Fig. S2A–D). While the latest observation is consistent with the expected tumor suppressive role of MRE11, its high expression observed in bad prognosis and MNA neuroblastoma suggests it might be required for tumor growth, in this subset. To test the functional role of MRE11 in neuroblastoma we knocked it down by shRNAi in the MYCN-repressible SHEP Tet21/N cell line^37^ (Fig. 1d), where we previously demonstrated MYCN transcriptionally regulates MRN complex expression^26^. Interestingly, this resulted in impaired proliferation and colony-forming ability in MYCN-overexpressing (henceforth MYCN+) cells but not in MYCN repressed (henceforth MYCN−) cells (Fig. 1e, f).

### The MRE11 inhibitor mirin impairs proliferation/survival in MNA neuroblastoma cells

**Fig. 2:**
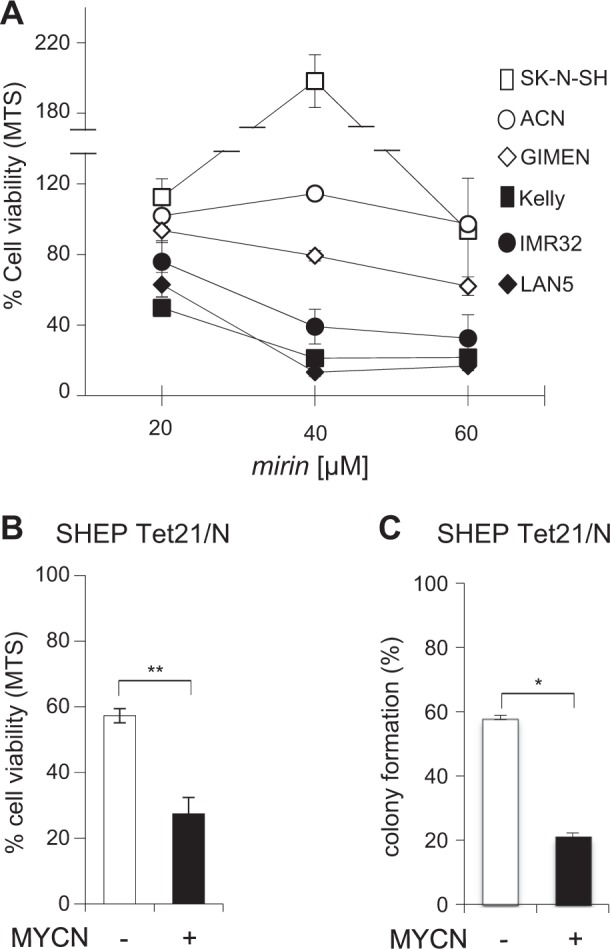
MNA neuroblastoma cells are sensitive to the MRE11 inhibitor mirin. **a**, **b** Effect of *mirin* treatment on cell viability of the indicated MNSC (open symbols) and MNA (black symbols) cells, and **b** MYCN+ and MYCN− SHEP Tet21/N cells, as measured by the MTS assay. **c** Effect of *mirin* treatment on colony formation assay in MYCN+ and MYCN− SHEP Tet21/N cells. For all panels, average data obtained by at least three independent experiments are reported as percentages compared to untreated controls, ±SD). *p-*Values were calculated by the Student’s *t*-test. *p < 0.05, **p < 0.01

To further explore whether MRE11 might be required for the growth of MYCN-driven tumors, we studied the effects of *mirin*, an extensively validated pharmacological inhibitor of MRE11 exonuclease activity^26,38,39^, by the MTS assay. MNSC neuroblastoma cells, a number of human non-neuroblastic cancer cell lines, and NIH3T3 cells showed essentially no or very modest inhibitory response to *mirin* up to 100 μM concentration (Fig. 2a and Table 1). In some instances, lower *mirin* concentrations (20–40 μM) even resulted in increased MTS assay values in MNSC models (i.e. SK-N-SH, A549). In sharp contrast, all MNA neuroblastoma cell lines showed a strong reduction in proliferation already at 40 μM concentration (Fig. 2a). Overall, *mirin* IC_50_ ranged between 22.81 and 48.16 μM, for MNA cell lines, and between 90 to 472 μM in all the others (Table 1). Importantly, *mirin* reduced cell viability and colony-forming ability more efficiently in MYCN+compared to MYCN− cells (Fig. 2b, c).

**Table 1:** Mirin IC_50_ in a panel of neuroblastoma and non-neuroblastoma cell lines

Cell line	IC50 (μM)
IMR32	48.16
KELLY	15.87
LAN5	22.81
SK-N-SH	128.3
GIMEN	89.95
HEK-293T	142.03
NIH3T3	472.41
A549	122.93
HepG2	92.23
Daoy	105.65

Overall, these data indicate that MRE11 is essential for proliferation and/or survival in MYCN-driven tumor cells.

### Mirin induces DNA damage and cell death in MNA cells

**Fig. 3:**
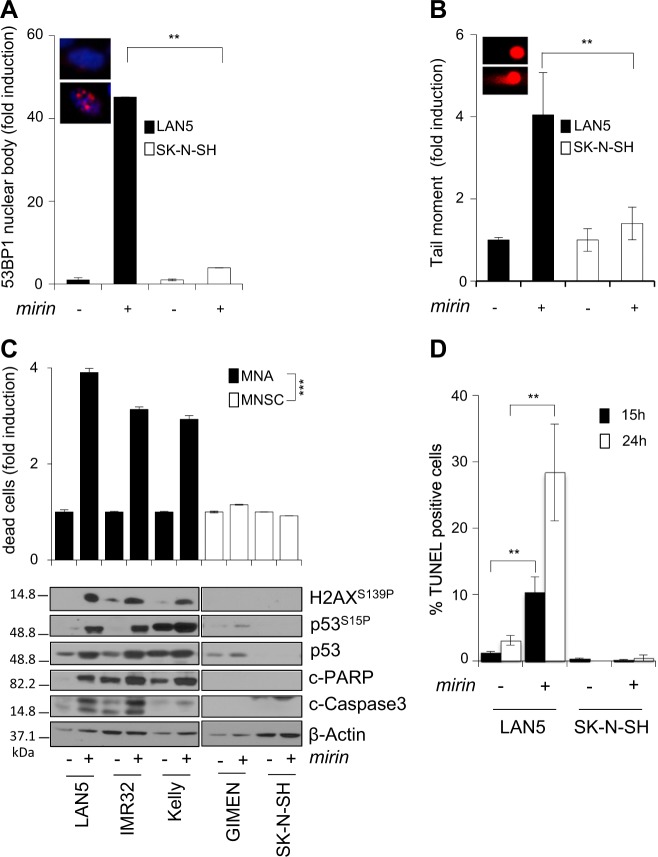
Mirin induces DNA damage, DDR, and cell death in MNA cell lines. **a**, **b** Percentage of cells showing >3 53BP1 nuclear bodies (**a**) and DNA damage as measured by the neutral comet assay (**b**), performed in LAN5 (MNA) and SK-N-SH (MNSC) cell lines treated with *mirin* or vehicle, for 5 h. For all panels, average data obtained by three independent experiments are reported as fold induction compared to untreated controls, ±SD). *p-*Values were calculated by the ANOVA test. **p < 0.01. Representative images of LAN5 cells treated with DMSO (above) or *mirin* (below) are given in the insets. **c** Evaluation of cell death and DDR in MNA (LAN5, IMR32, Kelly) and MNSC (GIMEN, SK-N-SH) cell lines, as measured by the trypan blue exclusion test (upper panel; average data obtained by three independent experiments are reported as fold induction compared to untreated controls, ±SD), and by WB analysis of the indicated proteins and phosphoepitopes (bottom panel), following *mirin* treatment. *p-*Values were calculated by the Student’s *t*-test. ***p < 0.001. **d** Percentage of TUNEL-positive apoptotic cells in LAN5 and SK-N-SH cell lines, following *mirin* treatment, for the indicated time points. Data obtained by three independent experiments are reported as percentage compared to untreated controls, ±SD. *p-*Values were calculated by the Student’s *t*-test. **p < 0.01

**Fig. 4:**
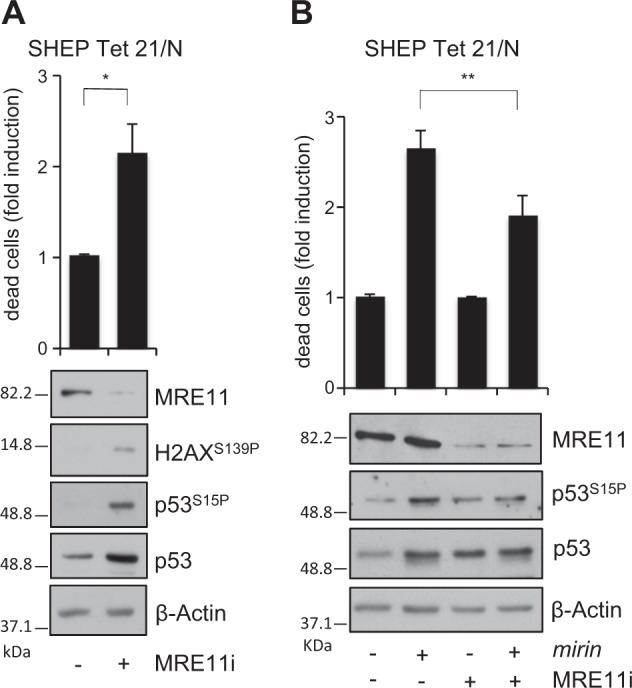
MRE11 depletion induces DDR and cell death and impairs mirin effects on MYCN+ cells. **a**, **b** MYCN + SHEP Tet21/N cells were transfected with no-target or MRE11-directed stealth RNAi. Cell death (upper panel) and DDR (bottom panel) were measured by the trypan blue exclusion assay and WB analysis of the indicated proteins and phosphoepitopes, respectively, after 24 h of transfection. Average data obtained by three independent cell death assays are reported as fold induction compared to untreated controls, ±SD. *p* was calculated by the Student’s *t*-test. *p < 0.05. **b** After MRE11 knockdown, the effects of *mirin* were assessed as above. *p* was calculated by the ANOVA test. **p < 0.01

The MRN complex is involved in multiple DNA repair pathways, including suppression of replication-associated DNA DSBs, most likely by preventing fork reversal and/or facilitating the restart of stalled replication forks^14–17^. Moreover, the MRN complex prevents the accumulation of DNA damages due to MYCN-dependent RS^26^. Consistently, *mirin* induced accumulation of 53BP1 nuclear bodies, a known marker of replication-associated DNA damage^40^, and DNA DSBs, in MNA but not MNSC cells (Fig. 3a, b; S3A, B). Furthermore, it induced H2AX and p53 phosphorylation in all MNA but not in MNSC cells (Fig. 3c), indicating the activation of a DDR. Early accumulation of DNA damage and DDR ended up in apoptotic cell death in MNA but not MNSC cells, as indicated by the trypan blue exclusion assay, expression of the cleaved forms of PARP1 and Caspase-3 and TUNEL staining (Fig. 3c, d). Similar to *mirin* treatment, also MRE11 depletion by RNAi induced H2AX and p53 phosphorylation and cell death, in MYCN+ cells (Fig. 4a). Moreover, an incomplete MRE11 knockdown reduced *mirin*-dependent p53 phosphorylation and accumulation and impaired *mirin*-induced cell death (Fig. 4b), supporting an “on target” activity of *mirin*.

Together with previously published data^41^, these observations suggest that MRE11 may be required to restrain the accumulation of MYCN-induced and replication-dependent DNA damage also in cancer cells.

### Mirin induces a p53-dependent cell death in MNA cells

**Fig. 5:**
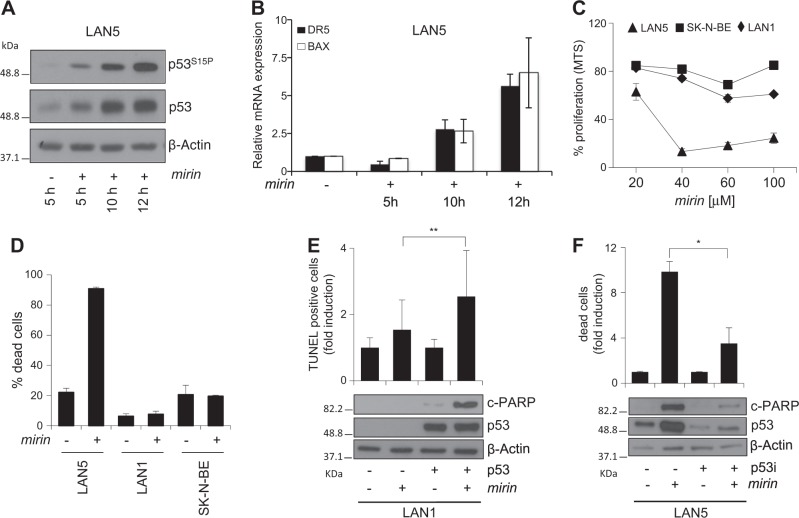
Mirin induces apoptosis through a p53-dependent mechanism in MNA cells. **a**, **b** WB analysis of the indicated proteins and phosphoepitopes (**a**) and real-time PCR quantification of the indicated transcripts (**b**), in LAN5 cells, following *mirin* treatment for the indicated time points. Blots were probed with β-actin as a loading control. Transcripts expression was normalized on GAPDH levels and reported as fold induction compared to untreated controls. Data obtained by three independent experiments are reported as means ± SD. **c**, **d** MTS assay (**c**) and trypan blue exclusion test (**d**) performed in p53 mutant (SK-N-BE), p53 null (LAN1), and p53 wild-type (LAN5) MNA neuroblastoma cell lines, after *mirin* treatment. **e** LAN1 cells were transiently transfected with a p53-expressing or an empty plasmid. Apoptosis was evaluated by TUNEL assay (upper panel) and by measuring the amount of the cleaved form of PARP1 (c-PARP) via WB (bottom panel), after 15 and 5 h of *mirin* treatment, respectively. Average data obtained by three independent TUNEL assays are reported as fold induction compared to controls, ±SD. *p* was calculated by the ANOVA test. **p < 0.01. **f** LAN5 cells transiently expressing either p53 shRNAi (p53i) or control shRNAi were evaluated for cell death by the trypan blue exclusion assay (upper panel) and by measuring the amount c-PARP via WB (bottom panel) after 15 and 5 h of *mirin* treatment, respectively. Average data obtained by three independent cell death assays are reported as fold induction compared to controls, ±SD. *p* was calculated by the ANOVA test. *p < 0.05

Of interest, *mirin*-dependent DDR activation was associated with an early and time-dependent accumulation of serine 15-phosphorylated p53 and with the early activation of pro-apoptotic p53 targets, like DR5 and BAX, in MNA cells (Fig. 5a, b), suggesting that *mirin*-induced cell death might depend on p53 activity. Consistently, *mirin* was unable to induce cell death in the p53-mutant SK-N-BE and in the p53-null LAN1 MNA cell lines (Fig. 5c, d). Moreover, exogenous p53 expression restored *mirin*-induced apoptotic cell death in LAN1 cells, whereas p53 depletion by shRNAi strongly reduced the apoptotic response to *mirin*, in LAN5 cells (Fig. 5e, f). Therefore, MRE11 inhibition by *mirin* is responsible for the DNA damage- and p53-dependent death, selectively occurring in MNA neuroblastoma cells.

### Mirin encapsulation in biocompatible polymeric nanocarriers

Due to its essential role in limiting the deleterious effect of RS in MYCN-driven cancer cells, MRE11 might represent a novel therapeutic target for MYCN-driven tumors. So far, *mirin* (and its structural derivatives^39^) is the unique pharmacological inhibitor of MRE11 and, unfortunately, is rather insoluble in aqueous, alcoholic, or oleic media, which strongly limits its use in vivo. To overcome this limitation and to facilitate *mirin* delivery into neuroblastoma xenografts in mice, we encapsulated the inhibitor in nanoparticles made of the copolymer poly(lactic-*co*-glycolic)-*co*-polyethylene glycol (PLGA-b-PEG) (Fig. S4A). This copolymer is biocompatible, biodegradable, and approved by the Food and Drug Administration and it has been exploited to create water-dispersible polymeric nanoparticles (PNPs), which can entrap, protect, and deliver lipophilic drugs within the body^42,43^. By using the nanoprecipitation technique^44^, *mirin* was efficiently encapsulated in PNPs, which are stable at physiological pH.

### Mirin reduces MNA tumor growth and induces DDR and apoptosis in vivo

**Fig. 6:**
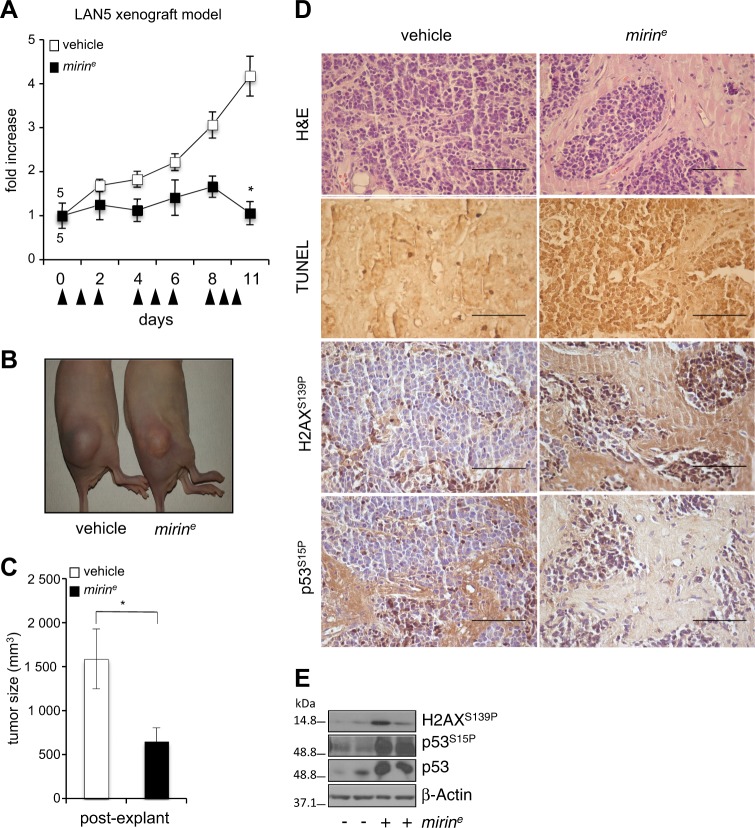
Encapsulated mirin reduces tumor growth and induces DDR and apoptosis in vivo. **a** Nude mice were subcutaneously inoculated with LAN5 cells. Mice were randomized and injected with 50 mg/kg of *mirin*^*e*^ or vehicle (empty nanoparticles) according to the indicated schedule (arrowheads), for a total of 1.25 mg of *mirin*^*e*^/mouse. Tumor growth was monitored with the caliper every other day. Data represent the fold increase of the mean tumor volume compared to day 0, for each treatment group (*n* = 5) ±SEM. **b** Representative images of xenografted mice at day 11, before tumor explant. **c** Graph represents the mean volume of the excised tumors ±SEM. *p* was calculated by the Student’s *t*-test. *p < 0.05. **d** Histology, TUNEL assay, and immunostaining for γH2AX and p53^S15P^ performed on tissue sections from tumor xenografts. Scale bar, 50 μm. **e** WB showing the expression level of the indicated proteins and phosphoepitopes in two representative tumors excised from *mirin*^*e*^- or vehicle-treated mice

First, we demonstrated that encapsulated *mirin* (*mirin*^e^) very efficiently induced DNA damage, DDR, and cell death in LAN5 cells, in vitro (Fig. S4B–D). Then, we tested it on LAN5 xenografts in nude mice. Neuroblastoma-xenografted mice were randomized and injected daily with *mirin*^e^ (50 mg/kg) or empty nanoparticles. With time, we observed a sharp suppression of tumor growth in *mirin*^e^-treated mice compared to controls (Fig. 6a–c). At the hystopathological level, *mirin*^e^ caused the appearance of hypocellular tumor areas with nests of neoplastic cells scattered in a desmoplastic stroma reaction, in which the occurrence of apoptosis was promptly detected by TUNEL staining (Fig. 6d). Moreover, *mirin*-induced DDR markers and p53 accumulation were detected in *mirin*-treated samples (Fig. 6d, e). These results indicate that MRE11 inhibition by *mirin* efficiently restrains tumor growth in vivo by inducing DDR and apoptosis.

## Discussion

**Fig. 7:**
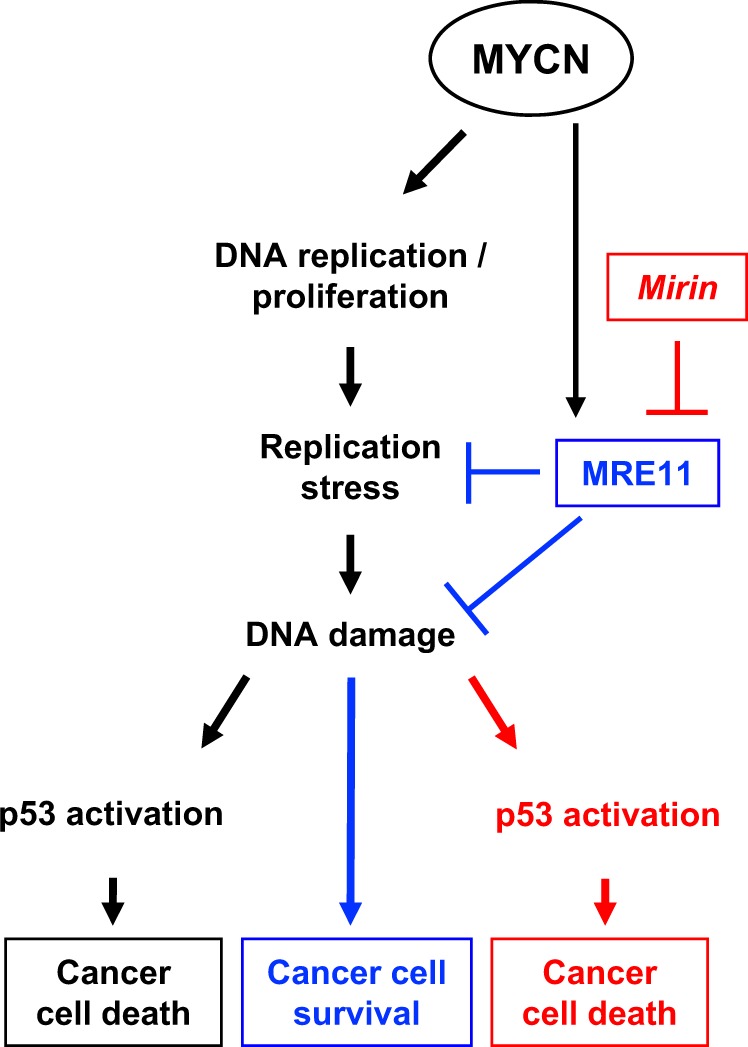
Schematic representation of the effects of MRE11 inhibition on MYCN-dependent tumors. While promoting an increased proliferation rate, MYCN overexpression also causes high levels of RS, which in turn would be responsible for the accumulation of DNA damage and cell death, if improperly controlled. However, MYCN regulates the expression of MRE11 to keep the deleterious effects of RS in check, thus allowing survival and proliferation of MYCN-driven tumor cells. Under these conditions, MRE11 inhibition leads to accumulation of RS-dependent DNA damage, DDR activation, and p53-dependent cell death

More effective and less toxic therapeutic approaches are urgently needed to overcome the poor prognosis of MYCN-driven tumors, such as high-risk/MNA neuroblastomas. Although inhibiting MYC functions proved to be an efficient anticancer strategy in preclinical models^45^, direct MYCN targeting in clinical settings has not been achieved, yet. Therefore, looking for MYCN-associated vulnerabilities might provide alternative strategies for the treatment of MYCN-driven tumors. Here, we have shown that targeting MRE11 leads to RS-dependent DNA damage accumulation, DDR, and p53-dependent cell death in MNA preclinical tumor models (Fig. 7), in vitro and in vivo.

Extensive literature supports that MYC oncogenes are strong inducers of RS^24,29,34^ and there is reason to believe that an appropriate control of RS needs to be enforced in cells proliferating under MYC pressure^26,36^. Indeed, we have recently shown that restraining MYCN-dependent RS is essential for cell proliferation and survival in the physiological context of MYCN-dependent expansion of cerebellar granule progenitor cells. To this end, MYCN transcriptionally controls the expression of the three components of the MRN complex to keep RS in-check during postnatal cerebellar development^41^. Since we noticed that high MRE11 expression occurs in MNA neuroblastoma, we speculated that MRE11 function might be required to control MYCN-associated RS and DNA damage, in this tumor subset. Consistently, its genetic or pharmacological inhibition led to accumulation of RS and DDR markers and caused cell death, specifically in MNA neuroblastoma.

Spehalski et al.^46^ reported that *Mre11* deficiency prevented tumorigenesis in mouse p53^−/−^ B-cell lymphoma models associated with oncogenic translocations involving *Myc* genes, indicating that MRE11 (and by extension the MRN complex) cannot be considered a standard tumor suppressor since its activity is required for cancer development. In line with this, we have now demonstrated that MRE11 is also required for an efficient RS control and for the survival of preclinical models of established MYCN-driven human cancer. Consistently, our data also add up that high MRE11 expression predicts very poor prognosis in high-risk neuroblastoma and that nanoparticle-mediated delivery of its pharmacological inhibitor *mirin* strongly impairs the growth of MNA tumor xenografts. Therefore, by addressing similar but not identical issues in different tumor models, all together these data strongly support the idea that targeting the MRN complex might represent a good strategy to tackle MYC(N)-driven tumors.

It has been recently reported that p53 orchestrates DNA replication restart homeostasis via a transcription- and apoptosis-independent function, by aiding MRE11 recruitment on stalled replication forks to prevent the activity of RAD52/Polθ mutagenic pathways^47^. While it is likely that MRE11 recruitment onto “stressed” forks is strictly connected to its tumor survival functions, data from Spehalski et al.^46^ imply that p53 is dispensable to this end, since they unveiled MRE11 oncogenic activity in p53^−^^/−^ B-cell lymphomas. Further studies will be definitely required to better define the complex relationships between p53 and the MRN complex at the replication forks, in normal and cancer cells. Nonetheless, our data clearly indicate that p53 is required to enhance cell death upon MRE11 inhibition, in MNA neuroblastoma, suggesting that preservation of p53 integral function is important in this context. Of relevance, p53 mutations rarely occur in primary neuroblastomas and in other MYCN-driven tumors^48,49^, supporting the possibility to enforce this therapeutic strategy in this tumor subset.

A large fraction of aggressive and MYCN-driven neuroblastomas is associated with a specific signature impinging on DNA replication, DNA repair, and cell cycle regulators^32,50^, suggesting that this gene expression program, which includes transcriptional regulation of MRE11 (ref.^26^), might be pivotal to increase cell tolerance to RS and its related DNA damages in MYCN-driven tumors. Based on the data presented here, these observations more broadly suggest that a number of potential candidates could be targeted for therapeutic purposes in MYCN-driven tumors. Moreover, this strategy could be quickly translated into clinical trials, due to the availability of approved drugs against well-known RS/DNA damage processing factors. In example, MYCN-driven neuroblastoma consistently showed a notable sensitivity to CHK1 and/or PARP inhibitors, in vitro and in xenograft models^51,52^. In addition, simultaneous targeting of DNA repair and cell cycle regulators seems to be most effective in killing MYCN-driven tumors by raising RS and DNA damage over the threshold for survival^51^. Nonetheless, the best candidates belonging to RS, DDR, or DNA repair pathways to be tackled in order to exploit MYCN-associated vulnerabilities are far from being fully established. Rather, there is room to believe that inhibiting distinct targets might differentially impact on tumor cell survival, in different molecular contexts. Indeed, while we described efficient killing of wild-type p53/MYCN-driven tumors by MRE11 inhibition, ATR-CHK1 signaling seems to be especially essential for MYC-driven tumors in a p53-defective backgrounds^34,35^, which rarely occurs in MNA neuroblastoma.

Remarkably, decreasing MRE11 expression may eventually favor proliferation and colony-forming capabilities in non-MNA cells (see Fig. 1e, f), which is consistent with very low MRE11 expression being associated with poor prognosis, in primary MNSC neuroblastomas. While these data match the renowned tumor suppressive function of MRE11, they also highlight that its inhibition might be detrimental in non-MYCN-driven tumors. These findings suggest the need for an appropriate tailoring of MRE11 inhibition and indicate the use of MNA and p53 status as potential selective biomarkers.

In conclusion, our work indicates that MRE11 inhibition might be an effective strategy to treat MYCN-driven tumors encouraging the search for new pharmacological inhibitors of the MRN complex.

## Materials and methods

### Public dataset gene expression analysis

R2-Genomics analysis and visualization platform (http://r2.amc.nl) was utilized to study gene expression of MRE11 in the SEQC-498-RPM dataset. Data were analyzed and downloaded from the website and formatted for publication.

### Cell lines and culture conditions

GIMEN were acquired from Banca Biologica and Cell Factory (Genoa, Italy; www.iclc.it); IMR32 and SK-N-BE from European Collection of Cell Cultures (Porton Down, UK; www.ecacc.org.uk); and LAN5 and Kelly from Deutsche Sammlung von Mikroorganismen und Zellkulturen (Braunschweig, Germany; www.dsmz.de). LAN1 and SK-N-SH cells were a kind gift of Dr. Nicole Gross (Department of Pediatrics, University Hospital, Lausanne, Switzerland) and Dr. Carol J. Thiele (NCI, Bethesda, MD), respectively. All these cell lines were validated by short tandem repeat DNA STR analysis (LGC Standards, Teddington, Middlesex, UK). SHEP Tet21/N cell line, received from Dr. Schwab, DKFZ, Heidelberg, Germany, is a MNSC neuroblastoma cell line in which exogenous *MYCN* expression is under the control of a tetracycline responsive promoter (Tet-off system)^37^. The cells were cultured and validated for MYCN induction as reported^37^. HEK-293T, NIH3T3, HepG2, DAOY, and A549 cells were obtained from the repository of our Department. All cells were grown in standard conditions as reported^53^ and tested for mycoplasma infection using EZ-PCR Mycoplasma Test Kit (Biological Industries, Cromwell, CT, USA).

Unless otherwise specified, cell lines were exposed to the MRE11 inhibitor *mirin* (Sigma Aldrich, St. Louis, MO, USA) at 40 μM concentration for either 15 (western blot analysis) or 48 h (trypan blue exclusion test and MTS assay).

### Transfection and lentivirus infection

MRE11 interference was performed using either a PLKO.1 plasmid expressing a specific shRNAi (CCGGACGGGAACGTCTGGGTAATTCCTCGAGGAATTACCCAGACGTTCCCGTTTTTTG; Sigma Aldrich) or by stealth RNAi (ACAUGUUGGUUUGCUGCGUAUUAAA; Invitrogen, Carlsbad, CA, USA). The p53 construct (pCAGp53) was previously described^27^. Plasmid and RNAi were transfected by Lipo2000 Transfection Reagent (Thermo Fisher Scientific, Waltham, MA, USA) according to the manufacturer’s instructions. p53 knockdown was achieved by infecting LAN5 cells with lentiviral transduction particles (pLV-WPXL-shp53 and pLV-WPXL-shCTR) as previously described^54^.

### RNA preparation and Q-PCR

mRNA was extracted using TRIzol reagent (Invitrogen) and quantitative reverse transcription-PCR was performed as previously described^55,56^. Q-PCR primer sequences are available as supplementary information.

### Western blot

Total protein extraction and western blot protocols have been previously described^56^. Immunoreactive bands were visualized by enhanced chemoluminescence (Advansta Inc., Menlo Park, CA, USA). Antibodies were as follows: MRE11 (12D7) ab214 (Abcam, Cambridge, UK); PARP 85 fragment #G7341 (Promega Corporation, Medison, WI, USA); Caspase-3 #9662, phospho-histone H2AX (ser 139) #2577 and phospho-p53 (Ser 15) #9284 (Cell Signaling Technology, Danvers, MA), USA; p53 (DO-1) #SC-126 and β-actin (I–19) #SC-1616 (Santa Cruz Biotechnology, INC, Dallas, TX, USA).

### Cell proliferation, cell death analysis, colony formation assay, and comet assay

MTS assay was performed using the MTS Cell Titer 96 aqueous one solution reagent (Promega Corporation) according to the manufacturer’s protocol and absorbance (450 nm) was recorded using a Glomax Multidetection Luminometer (Promega Corporation). Cell proliferation, cell death, and colony formation assays were performed as described^27,57^. For TUNEL staining, fixed cells were labeled using the in situ Cell Death Detection Kit (Roche Diagnostics, Indianapolis, IN, USA). At least 200 cells/sample were counted. Neutral comet assay was conducted as described^26^.

### Immunofluorescence assay and immunohistochemistry

Cells were fixed in 4% formaldehyde/phosphate-buffered saline (PBS) for 10 min at RT, permeabilized in 0.5% Triton X-100, blocked in 3% goat serum, in PBS and incubated with anti-53BP1 antibody (NB100-304, Novus Biological, LLC) for 1 h at room temperature and revealed with AlexaFluor secondary antibody (Life Technologies). Images were acquired on a LEICA DM 2500 microscope.

Formalin-fixed and paraffin-embedded tissue sections (4 μm thickness) were probed with phospho-histone H2AX (Ser 139) (20E3; Cell Signaling Technology), phospho-p53 (Ser 15) (#9284; Cell Signaling Technology) specific antibodies, according to the manufacturer's instruction of mouse2mouse HRP ready to use kit (MTM001, ScyTek Laboratories, Logan, UT, USA). Images were captured using the microscope Leica DM1000.

### Synthesis of mirin^e^

Poly (d,l-lactide-*co*-glycolide) (50/50) with the carboxylic acid end group (PLGA-COOH, inherent viscosity 0.12 dL/g, MW ~7 kDa) was purchased from Lakeshore Biomaterials (Birmingham, AL, USA). Polyethylene glycol with amino and carboxylic acid end groups (NH_2_-PEG-COOH, MW ~3 kDa) was purchased from Rapp Polymere GmbH (Tübingen, Germany). All aqueous solutions were prepared with deionized water obtained using an ultrafiltration system (Milli-Q, Millipore) with a measured resistivity above 18 MΩ/cm.

Two hundred and fifty milligrams of PLGA-*b*-PEG-COOH (10 kDa, 0.025 mmol) were prepared as previously described^58^ and 50 mg of *mirin* (0.23 mmol) were dissolved in 25 mL of DMSO. The organic phase was mixed to 250 mL of ultrapure water under vigorous stirring for 30 min, maintaining water/organic ratio 10/1 with a constant removal of the resulting solution. The mixture was subsequently purified and concentrated using centrifugal filter devices (Amicon Ultra, Ultracell membrane with 100,000 NMWL; Millipore, Billerica, MA, USA) to a final volume of 20 mL, passed through a filter SterivexTM-GP 0.22 μm of polyether sulfone (Millipore, USA) and stored at 4 °C. The same procedure was exploited to obtain empty nanoparticles. DLS measurements were performed on a Malvern Zetasizernano-S working with a 532 nm laser beam. ζ potential measurements were conducted in DTS1060C-Clear disposable zeta cells at 25 °C. To asses *mirin* concentration, a sample aliquot (50 µL) was diluted with 3 mL of DMSO and analyzed by UV–Vis (*λ*_max_ = 453 nm) after sonication (15 min) in order to destroy the nanoparticles and re-disperse the drug.

### Xenograft generation and analysis

LAN5 cells (2.5 × 10^6^ cells/flank) were suspended in an equal volume of medium and Matrigel (BD Biosciences, Heidelberg, Germany) and injected at the posterior flank of female BALB/c nude mice (nu/nu) (Charles River Laboratories, Lecco, Italy). When tumors reached a median size of 260 mm^3^, animals were randomly divided into two groups (*n* = 5) and locally injected with vehicle or *mirin*^*e*^ for 11days. Tumor growth was monitored by caliper. Tumor volumes change was calculated by the formula length × width × 0.5 × (length + width). Animal experiment was approved by local ethics authorities (protocol no. 379/2016-PR) and was performed according to the guidelines for animal care.

### Statistical analysis

Statistical analysis was performed by a standard two-tailed Student’s *t*-test or ANOVA test using GraphPRISM6 software, as indicated. For all tests a *p*-value <0.05 was considered to indicate statistical significant differences. The software GraphPRISM6 was used to determine IC_50_. Error bar represent standard deviation (SD) or standard errors (SEM) as indicated.

## Electronic supplementary material

Supplemental material

## Footnotes

1 Data refer to the GSE62564 dataset (498 neuroblastoma samples). Although not reported here, similar results can also be obtained by interrogating GSE45547 and GSE3960 datasets (649 and 101 neuroblastoma samples, respectively).
