# Supplementary material for: MRE11 inhibition highlights a replication stress-dependent vulnerability of MYCN-driven tumors
Source: Cell Death Dis. 2018 Aug 30;9(9):895. doi: 10.1038/s41419-018-0924-z (PMC6117286; doi:10.1038/s41419-018-0924-z)
Supplement: Supplementary file 1 — Supplemental material [file 41419_2018_924_MOESM1_ESM.pptx]

## Slide 1
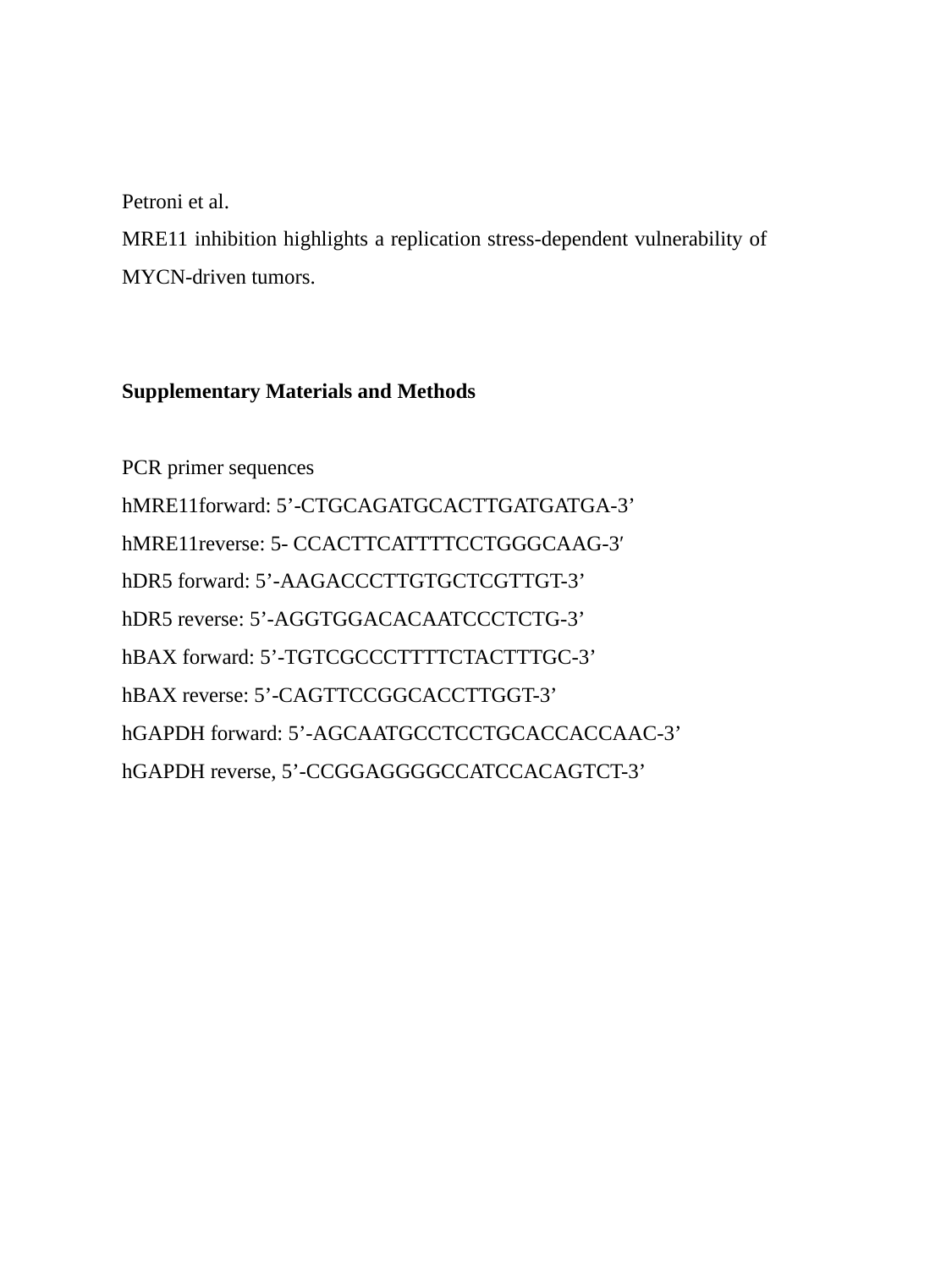

Petroni et al.
MRE11 inhibition highlights a replication stress-dependent vulnerability of MYCN-driven tumors.
Supplementary Materials and Methods
PCR primer sequences
hMRE11forward: 5’-CTGCAGATGCACTTGATGATGA-3’
hMRE11reverse: 5- CCACTTCATTTTCCTGGGCAAG-3′
hDR5 forward: 5’-AAGACCCTTGTGCTCGTTGT-3’
hDR5 reverse: 5’-AGGTGGACACAATCCCTCTG-3’
hBAX forward: 5’-TGTCGCCCTTTTCTACTTTGC-3’
hBAX reverse: 5’-CAGTTCCGGCACCTTGGT-3’
hGAPDH forward: 5’-AGCAATGCCTCCTGCACCACCAAC-3’
hGAPDH reverse, 5’-CCGGAGGGGCCATCCACAGTCT-3’

## Slide 2
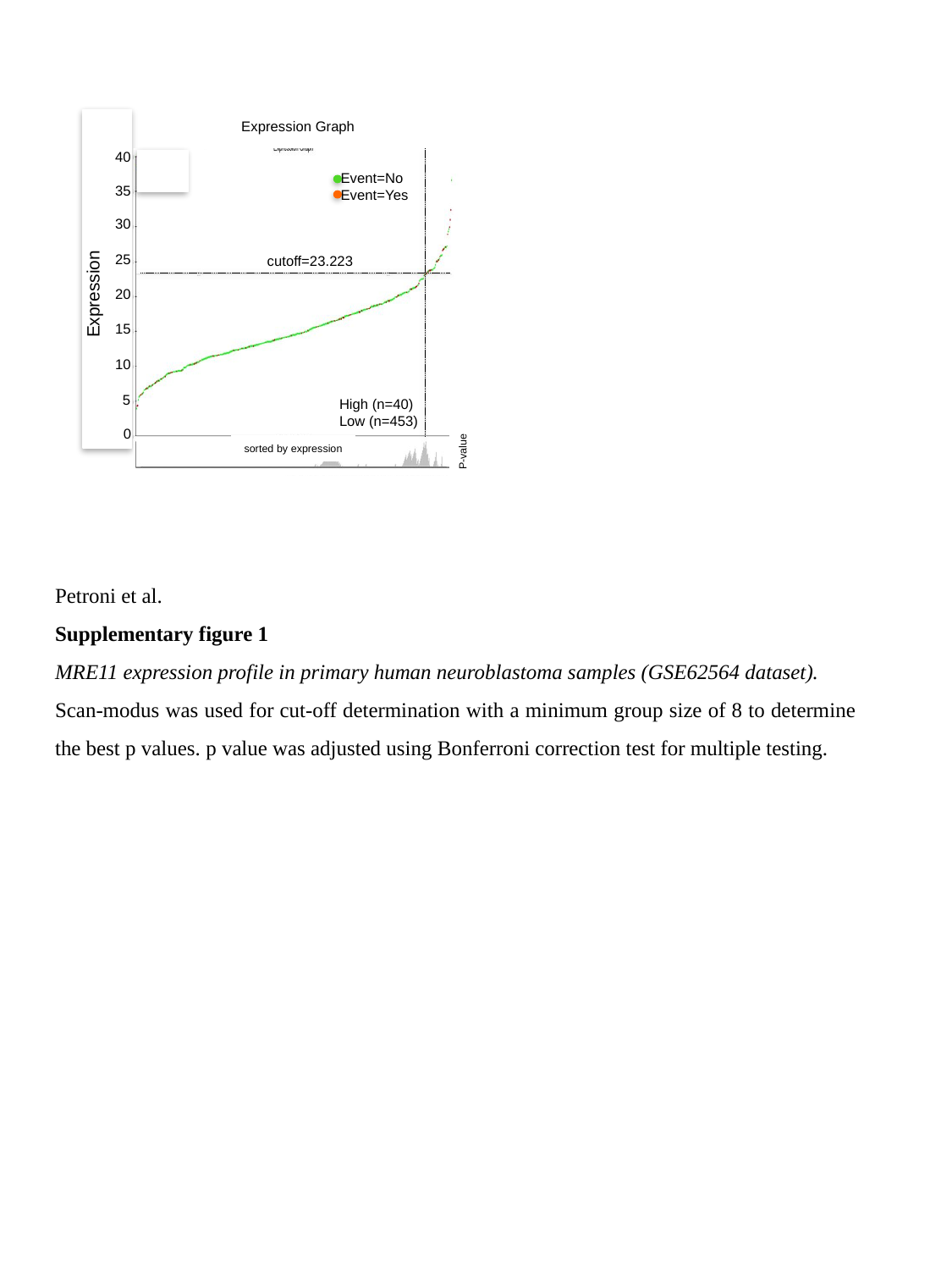

### Chart
| Category | |
|---|---|
Expression Graph
40
Event=No
Event=Yes
35
30
25
cutoff=23.223
Expression
20
15
10
5
High (n=40)
Low (n=453)
0
sorted by expression
P-value
Petroni et al.
Supplementary figure 1
MRE11 expression profile in primary human neuroblastoma samples (GSE62564 dataset).
Scan-modus was used for cut-off determination with a minimum group size of 8 to determine the best p values. p value was adjusted using Bonferroni correction test for multiple testing.

## Slide 3
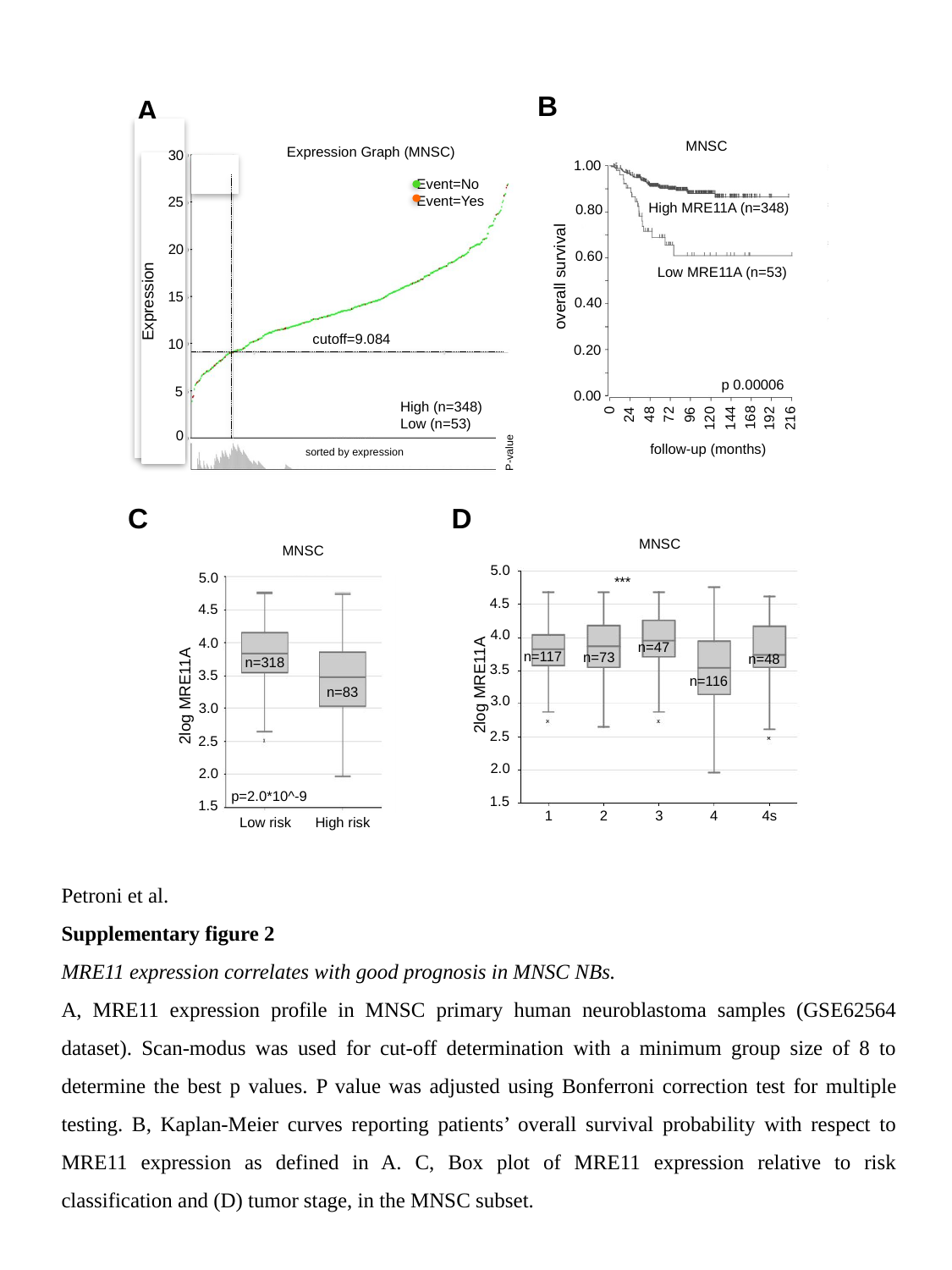

### Chart
| Category | |
|---|---|B
A
1.00
0.80
High MRE11A (n=348)
0.60
Low MRE11A (n=53)
overall survival
0.40
0.20
p 0.00006
0.00
0
96
48
24
72
192
216
168
120
144
follow-up (months)
MNSC
Expression Graph (MNSC)
30
Event=No
Event=Yes
25
20
15
Expression
cutoff=9.084
10
5
High (n=348)
Low (n=53)
0
sorted by expression
P-value
C
D
MNSC
5.0
4.5
4.0
3.5
2log MRE11A
3.0
2.5
2.0
1.5
1
2
3
4
4s
n=47
n=117
n=73
n=48
n=116
MNSC
5.0
4.5
4.0
3.5
2log MRE11A
3.0
2.5
2.0
1.5
Low risk
High risk
n=318
n=83
p=2.0*10^-9
***
Petroni et al.
Supplementary figure 2
MRE11 expression correlates with good prognosis in MNSC NBs.
A, MRE11 expression profile in MNSC primary human neuroblastoma samples (GSE62564 dataset). Scan-modus was used for cut-off determination with a minimum group size of 8 to determine the best p values. P value was adjusted using Bonferroni correction test for multiple testing. B, Kaplan-Meier curves reporting patients’ overall survival probability with respect to MRE11 expression as defined in A. C, Box plot of MRE11 expression relative to risk classification and (D) tumor stage, in the MNSC subset.

## Slide 4
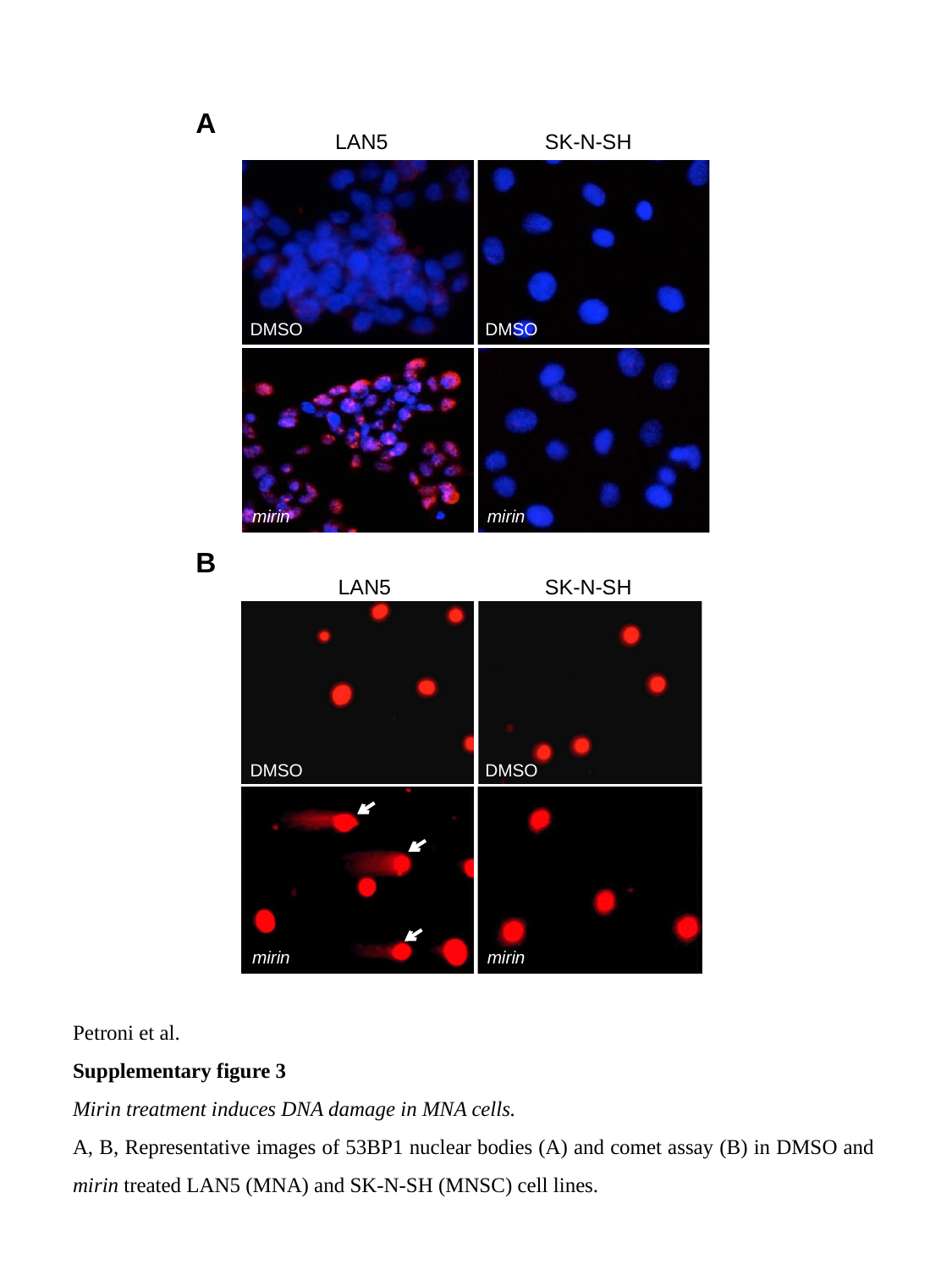

A
LAN5
SK-N-SH
DMSO
DMSO
mirin
mirin
B
LAN5
SK-N-SH
DMSO
DMSO
mirin
mirin
Petroni et al.
Supplementary figure 3
Mirin treatment induces DNA damage in MNA cells.
A, B, Representative images of 53BP1 nuclear bodies (A) and comet assay (B) in DMSO and mirin treated LAN5 (MNA) and SK-N-SH (MNSC) cell lines.

## Slide 5
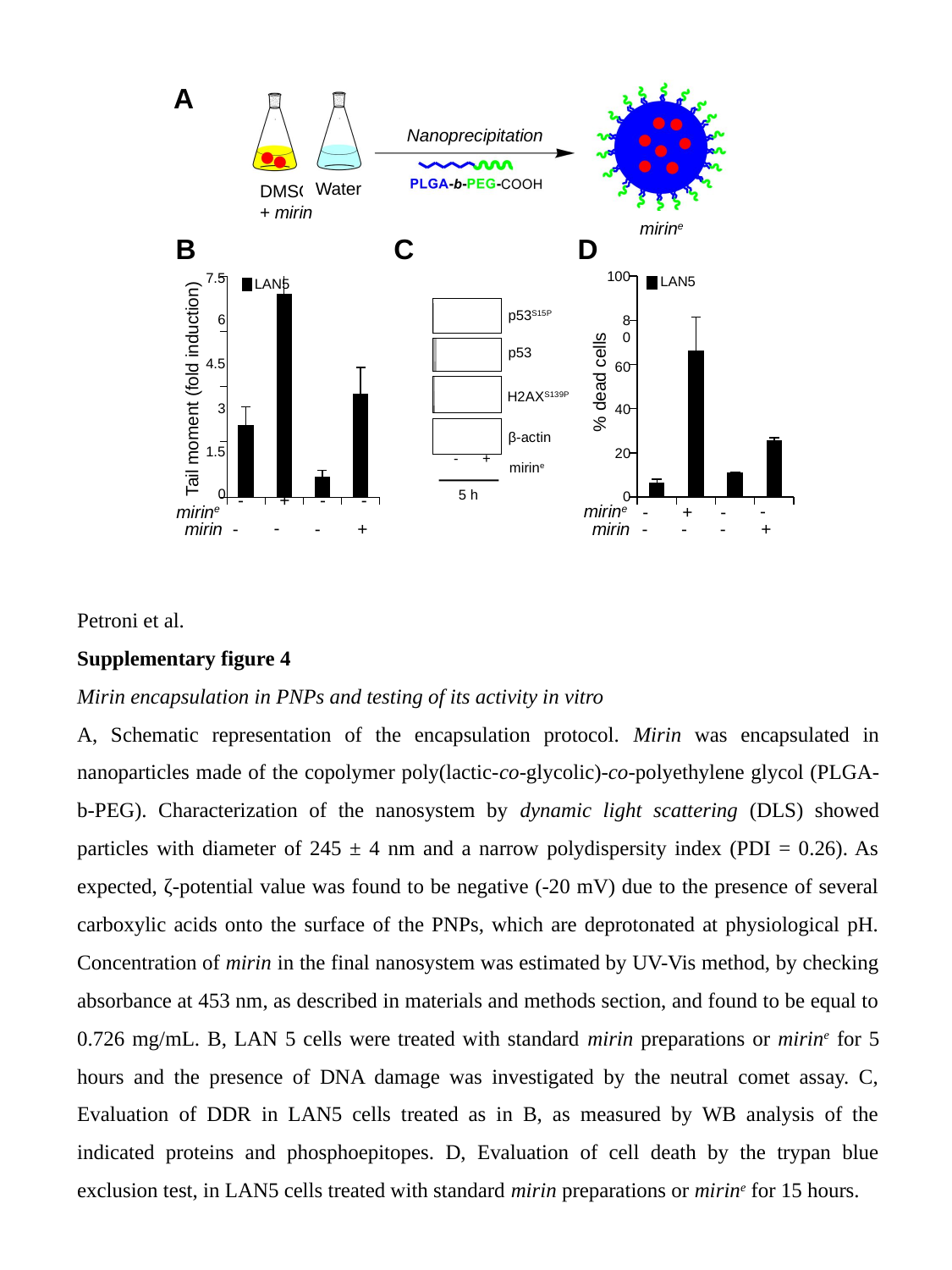

A
Nanoprecipitation
Water
DMSO
+ mirin
mirine
B
C
D
### Chart
| Category | |
|---|---|LAN5
100
80
60
% dead cells
40
20
 -
 -
 +
 -
0
 -
 +
 -
 -
mirin
### Chart
| Category | |
|---|---|LAN5
7.5
6
4.5
Tail moment (fold induction)
3
1.5
 -
 +
 -
 -
0
mirine
mirin
 +
 -
 -
 -
p53S15P
p53
H2AXS139P
β-actin
mirine
 -
 +
5 h
mirine
Petroni et al.
Supplementary figure 4
Mirin encapsulation in PNPs and testing of its activity in vitro
A, Schematic representation of the encapsulation protocol. Mirin was encapsulated in nanoparticles made of the copolymer poly(lactic-co-glycolic)-co-polyethylene glycol (PLGA-b-PEG). Characterization of the nanosystem by dynamic light scattering (DLS) showed particles with diameter of 245 ± 4 nm and a narrow polydispersity index (PDI = 0.26). As expected, ζ-potential value was found to be negative (-20 mV) due to the presence of several carboxylic acids onto the surface of the PNPs, which are deprotonated at physiological pH. Concentration of mirin in the final nanosystem was estimated by UV-Vis method, by checking absorbance at 453 nm, as described in materials and methods section, and found to be equal to 0.726 mg/mL. B, LAN 5 cells were treated with standard mirin preparations or mirine for 5 hours and the presence of DNA damage was investigated by the neutral comet assay. C, Evaluation of DDR in LAN5 cells treated as in B, as measured by WB analysis of the indicated proteins and phosphoepitopes. D, Evaluation of cell death by the trypan blue exclusion test, in LAN5 cells treated with standard mirin preparations or mirine for 15 hours.

## Slide 6
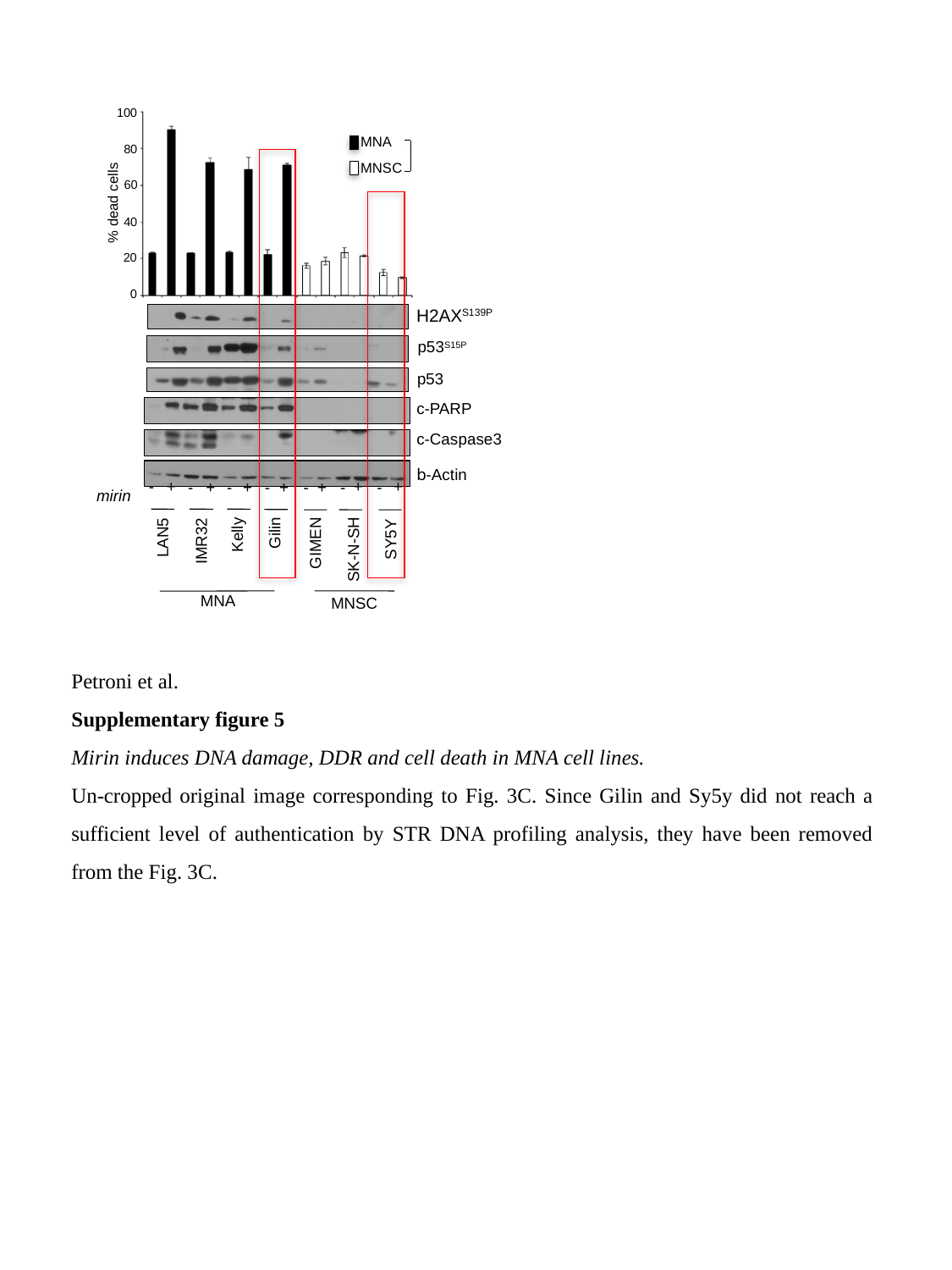

100
80
60
40
20
0
% dead cells
MNA
MNSC
H2AXS139P
p53S15P
p53
c-PARP
c-Caspase3
b-Actin
 -
 +
 +
 +
 +
 +
 +
 -
 -
 -
 -
 +
 -
 -
mirin
Gilin
Kelly
SY5Y
LAN5
IMR32
GIMEN
SK-N-SH
MNA
MNSC
Petroni et al.
Supplementary figure 5
Mirin induces DNA damage, DDR and cell death in MNA cell lines.
Un-cropped original image corresponding to Fig. 3C. Since Gilin and Sy5y did not reach a sufficient level of authentication by STR DNA profiling analysis, they have been removed from the Fig. 3C.
